# 3-(Benzimidazolium-2-yl)propionate dihydrate

**DOI:** 10.1107/S1600536808031346

**Published:** 2008-10-04

**Authors:** Xin Xiao, Yun-Qiang Zhang, Sai-Feng Xue, Zhu Tao

**Affiliations:** a Key Laboratory of Macrocyclic and Supramolecular Chemistry of Guizhou Province, Guizhou University, Guiyang 550025, People’s Republic of China; b Institute of Applied Chemistry, Guizhou University, Guiyang 550025, People’s Republic of China

## Abstract

In the crystal struture of the title compound, C_10_H_10_N_2_O_2_·2H_2_O, the component species are linked to the water mol­ecules by N—H⋯O and O—H⋯O hydrogen bonds to form a three-dimensional network structure.

## Related literature

For general background, see: Day & Arnold (2000 ▶); Day *et al.* (2002 ▶); Freeman *et al.* (1981 ▶); Kim *et al.* (2000 ▶). For related structure, see: Ge *et al.* (2007 ▶).
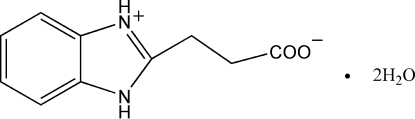


## Experimental

### 

#### Crystal data

C_10_H_10_N_2_O_2_·2H_2_O
*M* *_r_* = 226.23
Monoclinic, 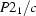

*a* = 18.444 (3) Å
*b* = 4.9730 (8) Å
*c* = 11.9097 (19) Å
β = 94.530 (5)°
*V* = 1089.0 (3) Å^3^
*Z* = 4
Mo *K*α radiation
μ = 0.11 mm^−1^
*T* = 273 (2) K
0.29 × 0.26 × 0.20 mm

#### Data collection

Bruker SMART APEXII CCD area-detector diffractometer
Absorption correction: none
7787 measured reflections
2002 independent reflections
1394 reflections with *I* > 2σ(*I*)
*R*_int_ = 0.066

#### Refinement

*R*[*F* ^2^ > 2σ(*F* ^2^)] = 0.047
*wR*(*F* ^2^) = 0.113
*S* = 1.08
1971 reflections
161 parameters
6 restraints
H atoms treated by a mixture of independent and constrained refinement
Δρ_max_ = 0.19 e Å^−3^
Δρ_min_ = −0.23 e Å^−3^

### 

Data collection: *APEX2* (Bruker, 2004 ▶); cell refinement: *SAINT* (Bruker, 2004 ▶); data reduction: *SAINT*; program(s) used to solve structure: *SHELXS97* (Sheldrick, 2008 ▶); program(s) used to refine structure: *SHELXL97* (Sheldrick, 2008 ▶); molecular graphics: *ORTEP-3 for Windows* (Farrugia, 1997 ▶); software used to prepare material for publication: *WinGX* (Farrugia, 1999 ▶).

## Supplementary Material

Crystal structure: contains datablocks global, I. DOI: 10.1107/S1600536808031346/ng2490sup1.cif

Structure factors: contains datablocks I. DOI: 10.1107/S1600536808031346/ng2490Isup2.hkl

Additional supplementary materials:  crystallographic information; 3D view; checkCIF report

## Figures and Tables

**Table 1:** Hydrogen-bond geometry (Å, °)

*D*—H⋯*A*	*D*—H	H⋯*A*	*D*⋯*A*	*D*—H⋯*A*
N1—H1N⋯O1^i^	0.86	1.86	2.700 (2)	166
N2—H2N⋯O2^ii^	0.86	1.80	2.654 (3)	170
O2*W*—H2*W*1⋯O1*W*^iii^	0.841 (17)	2.007 (18)	2.842 (3)	172 (3)

Symmetry codes: (i) 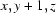; (ii) 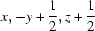; (iii) 
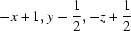
.

**Fig. 1.**
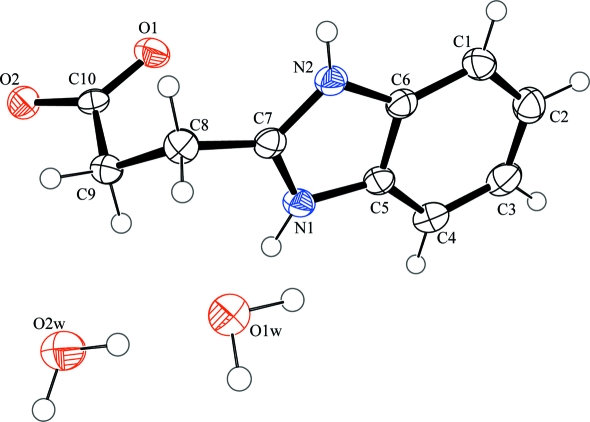
The molecular structure of (I) showing the atom-labelling scheme. Displacement ellipsoids are drawn at the 50% probability level.

## supplementary crystallographic information

### Comment

As part of our ongoing investigation on benzimidazole compounds, we present a compound containing multiple functional groups that can develop strong intermolecular interactions with cucurbit[*n*]urils (CB[*n*]) (Freeman *et al.*, 1981; Day & Arnold, 2000; Day *et al.*, 2002; Kim *et al.*, 2000; Ge *et al.*, 2007).

The crystal structure of the title compound (Fig. 1) consists of a 3-(1*H*-benzo[*d*]imidazol-2-yl) propanoic acid organic molecule and two lattice water molecules. the dihedral angle between the benzene ring (C1,C2,C3,C4,C5,C6) and the imidazole ring (C5,C6,C9,N2,C7,N1) is 0.61 (13)°. The C7—C8—C9—C10 torsion angle is -66.3 (3)°. The title compound forms intermolecular H bonds whereas the protonated N1 and N2 atoms act as hydrogen-bond donors and the O1 and O2 atoms act as hydrogen-bond acceptors, the O—H···O hydrogen bonds are also observed between the water molecules O2W and O1W (Table 1). these contacts and the cross-linking interactions stabilize the crystal packing.

### Experimental

The propionic anhydride (13 g, 0.1 mol) was dissolved in hot water (100 ml) with stirring, and a warm solution of 1,2-diaminobenzene(10.8 g, 0.1 mol) in 1,4-dioxane (100 ml) was added, following by the addition of ployphosphoric acid (50 ml) as catalyst. The mixture was refluxed for 8 h and then cooled, the solution was filtered and the filtrate was set aside for three weeks to obtain colorless crystals.

### Refinement

Water H atoms were located in a difference Fourier map and refined as riding in their as-found positions relative to O atoms with *U*_iso_(H) = 1.2*U*_eq_(O). All other H atoms were placed in calculated positions and refined as riding, with C—H = 0.93–0.97 Å, N—H = 0.86 Å, and *U*_iso_(H) = 1.2–1.5 *U*_eq_(C,*N*).

### Crystal data

**Table:**

C_10_H_10_N_2_O_2_·2H_2_O	*F*(000) = 480
*M**_r_* = 226.23	*D*_x_ = 1.380 Mg m^−^^3^
Monoclinic, *P*2_1_/*c*	Mo *K*α radiation, λ = 0.71073 Å
Hall symbol: -P 2ybc	Cell parameters from 2002 reflections
*a* = 18.444 (3) Å	θ = 1.1–25.4°
*b* = 4.9730 (8) Å	µ = 0.11 mm^−^^1^
*c* = 11.9097 (19) Å	*T* = 273 K
β = 94.530 (5)°	Prism, colourless
*V* = 1089.0 (3) Å^3^	0.29 × 0.26 × 0.20 mm
*Z* = 4	

### Data collection

**Table:**

Bruker CCD area-detector diffractometer	1394 reflections with *I* > 2σ(*I*)
Radiation source: fine-focus sealed tube	*R*_int_ = 0.066
graphite	θ_max_ = 25.4°, θ_min_ = 1.1°
φ and ω scans	*h* = −21→19
7787 measured reflections	*k* = −5→4
2002 independent reflections	*l* = −13→14

### Refinement

**Table:**

Refinement on *F*^2^	Primary atom site location: structure-invariant direct methods
Least-squares matrix: full	Secondary atom site location: difference Fourier map
*R*[*F*^2^ > 2σ(*F*^2^)] = 0.047	Hydrogen site location: inferred from neighbouring sites
*wR*(*F*^2^) = 0.113	H atoms treated by a mixture of independent and constrained refinement
*S* = 1.08	*w* = 1/[σ^2^(*F*_o_^2^) + (0.0354*P*)^2^ + 0.5171*P*] where *P* = (*F*_o_^2^ + 2*F*_c_^2^)/3
1971 reflections	(Δ/σ)_max_ < 0.001
161 parameters	Δρ_max_ = 0.19 e Å^−^^3^
6 restraints	Δρ_min_ = −0.23 e Å^−^^3^

### Special details

**Table:**

Geometry. All e.s.d.'s (except the e.s.d. in the dihedral angle between two l.s. planes) are estimated using the full covariance matrix. The cell e.s.d.'s are taken into account individually in the estimation of e.s.d.'s in distances, angles and torsion angles; correlations between e.s.d.'s in cell parameters are only used when they are defined by crystal symmetry. An approximate (isotropic) treatment of cell e.s.d.'s is used for estimating e.s.d.'s involving l.s. planes.
Refinement. Refinement of *F*^2^ against ALL reflections. The weighted *R*-factor *wR* and goodness of fit *S* are based on *F*^2^, conventional *R*-factors *R* are based on *F*, with *F* set to zero for negative *F*^2^. The threshold expression of *F*^2^ > σ(*F*^2^) is used only for calculating *R*-factors(gt) *etc*. and is not relevant to the choice of reflections for refinement. *R*-factors based on *F*^2^ are statistically about twice as large as those based on *F*, and *R*- factors based on ALL data will be even larger.

### Fractional atomic coordinates and isotropic or equivalent isotropic displacement parameters (Å^2^)

**Table:**

	*x*	*y*	*z*	*U*_iso_*/*U*_eq_	
O1	0.24941 (9)	0.3021 (3)	0.56604 (13)	0.0306 (5)	
O2	0.31408 (9)	0.3485 (3)	0.41718 (12)	0.0285 (4)	
O1W	0.45721 (11)	0.3587 (4)	0.36594 (15)	0.0360 (5)	
O2W	0.52898 (12)	−0.1307 (4)	0.37002 (17)	0.0398 (5)	
N1	0.22255 (10)	0.8913 (4)	0.70511 (15)	0.0249 (5)	
H1N	0.2296	1.0021	0.6516	0.030*	
N2	0.23986 (10)	0.5625 (4)	0.82389 (15)	0.0241 (5)	
H2N	0.2595	0.4266	0.8591	0.029*	
C1	0.12302 (14)	0.6171 (5)	0.92071 (19)	0.0290 (6)	
H1A	0.1303	0.4775	0.9723	0.035*	
C2	0.06132 (14)	0.7751 (5)	0.9168 (2)	0.0331 (7)	
H2A	0.0264	0.7417	0.9673	0.040*	
C3	0.04988 (14)	0.9848 (5)	0.8385 (2)	0.0334 (7)	
H3A	0.0074	1.0858	0.8380	0.040*	
C4	0.09993 (13)	1.0447 (5)	0.7624 (2)	0.0300 (6)	
H4A	0.0924	1.1838	0.7107	0.036*	
C5	0.16226 (13)	0.8870 (5)	0.76689 (18)	0.0237 (6)	
C6	0.17353 (13)	0.6765 (5)	0.84417 (18)	0.0238 (6)	
C7	0.26826 (13)	0.6960 (5)	0.74162 (18)	0.0234 (6)	
C8	0.34047 (13)	0.6448 (5)	0.69974 (18)	0.0264 (6)	
H8A	0.3582	0.4724	0.7285	0.032*	
H8B	0.3741	0.7816	0.7299	0.032*	
C9	0.34097 (13)	0.6433 (5)	0.57180 (18)	0.0252 (6)	
H9A	0.3207	0.8115	0.5425	0.030*	
H9B	0.3909	0.6334	0.5521	0.030*	
C10	0.29840 (13)	0.4120 (5)	0.51556 (18)	0.0227 (6)	
H1W1	0.4133 (12)	0.352 (7)	0.386 (3)	0.084 (13)*	
H1W2	0.4755 (16)	0.208 (4)	0.384 (3)	0.059 (11)*	
H2W1	0.5341 (16)	−0.149 (6)	0.3009 (16)	0.053 (9)*	
H2W2	0.5073 (19)	−0.268 (5)	0.387 (3)	0.089 (14)*	

### Atomic displacement parameters (Å^2^)

**Table:**

	*U*^11^	*U*^22^	*U*^33^	*U*^12^	*U*^13^	*U*^23^
O1	0.0340 (10)	0.0248 (10)	0.0334 (9)	−0.0060 (8)	0.0054 (8)	0.0027 (8)
O2	0.0334 (10)	0.0268 (10)	0.0255 (8)	−0.0038 (8)	0.0042 (8)	−0.0056 (7)
O1W	0.0374 (13)	0.0303 (12)	0.0416 (11)	−0.0013 (10)	0.0106 (10)	−0.0001 (9)
O2W	0.0486 (13)	0.0315 (12)	0.0399 (11)	−0.0038 (11)	0.0079 (10)	−0.0010 (10)
N1	0.0311 (12)	0.0209 (12)	0.0230 (9)	−0.0003 (10)	0.0032 (9)	0.0034 (9)
N2	0.0290 (12)	0.0205 (12)	0.0225 (9)	0.0022 (10)	0.0013 (9)	0.0013 (8)
C1	0.0336 (15)	0.0257 (15)	0.0281 (12)	−0.0059 (13)	0.0043 (11)	−0.0011 (11)
C2	0.0315 (15)	0.0340 (16)	0.0348 (14)	−0.0053 (13)	0.0090 (12)	−0.0058 (12)
C3	0.0292 (15)	0.0272 (15)	0.0438 (15)	0.0027 (13)	0.0035 (13)	−0.0062 (13)
C4	0.0309 (15)	0.0231 (15)	0.0354 (13)	0.0009 (12)	−0.0006 (12)	−0.0010 (11)
C5	0.0267 (14)	0.0194 (14)	0.0248 (11)	−0.0044 (11)	0.0016 (11)	−0.0032 (10)
C6	0.0268 (14)	0.0198 (13)	0.0248 (12)	−0.0005 (11)	0.0010 (11)	−0.0029 (10)
C7	0.0289 (14)	0.0202 (13)	0.0206 (11)	−0.0035 (12)	−0.0004 (10)	−0.0045 (10)
C8	0.0240 (14)	0.0271 (15)	0.0279 (12)	−0.0009 (12)	0.0006 (11)	−0.0011 (11)
C9	0.0289 (14)	0.0208 (14)	0.0264 (12)	−0.0028 (12)	0.0049 (11)	0.0005 (11)
C10	0.0257 (14)	0.0167 (13)	0.0256 (12)	0.0048 (11)	0.0012 (11)	0.0050 (10)

### Geometric parameters (Å, °)

**Table:**

O1—C10	1.250 (3)	C2—C3	1.404 (4)
O2—C10	1.269 (3)	C2—H2A	0.9300
O1W—H1W1	0.863 (18)	C3—C4	1.376 (3)
O1W—H1W2	0.845 (18)	C3—H3A	0.9300
O2W—H2W1	0.841 (17)	C4—C5	1.389 (3)
O2W—H2W2	0.823 (18)	C4—H4A	0.9300
N1—C7	1.336 (3)	C5—C6	1.399 (3)
N1—C5	1.381 (3)	C7—C8	1.481 (3)
N1—H1N	0.8600	C8—C9	1.525 (3)
N2—C7	1.325 (3)	C8—H8A	0.9700
N2—C6	1.387 (3)	C8—H8B	0.9700
N2—H2N	0.8600	C9—C10	1.518 (3)
C1—C2	1.380 (3)	C9—H9A	0.9700
C1—C6	1.386 (3)	C9—H9B	0.9700
C1—H1A	0.9300		
			
H1W1—O1W—H1W2	105 (3)	C4—C5—C6	121.8 (2)
H2W1—O2W—H2W2	104 (3)	C1—C6—N2	132.5 (2)
C7—N1—C5	109.22 (19)	C1—C6—C5	121.3 (2)
C7—N1—H1N	125.4	N2—C6—C5	106.2 (2)
C5—N1—H1N	125.4	N2—C7—N1	109.2 (2)
C7—N2—C6	109.2 (2)	N2—C7—C8	125.6 (2)
C7—N2—H2N	125.4	N1—C7—C8	125.2 (2)
C6—N2—H2N	125.4	C7—C8—C9	114.5 (2)
C2—C1—C6	116.9 (2)	C7—C8—H8A	108.6
C2—C1—H1A	121.6	C9—C8—H8A	108.6
C6—C1—H1A	121.6	C7—C8—H8B	108.6
C1—C2—C3	121.7 (2)	C9—C8—H8B	108.6
C1—C2—H2A	119.2	H8A—C8—H8B	107.6
C3—C2—H2A	119.2	C10—C9—C8	113.63 (19)
C4—C3—C2	121.6 (2)	C10—C9—H9A	108.8
C4—C3—H3A	119.2	C8—C9—H9A	108.8
C2—C3—H3A	119.2	C10—C9—H9B	108.8
C3—C4—C5	116.7 (2)	C8—C9—H9B	108.8
C3—C4—H4A	121.7	H9A—C9—H9B	107.7
C5—C4—H4A	121.7	O1—C10—O2	124.2 (2)
N1—C5—C4	132.0 (2)	O1—C10—C9	119.2 (2)
N1—C5—C6	106.1 (2)	O2—C10—C9	116.6 (2)
			
C6—C1—C2—C3	0.5 (4)	C4—C5—C6—C1	−0.5 (3)
C1—C2—C3—C4	−0.6 (4)	N1—C5—C6—N2	−0.7 (2)
C2—C3—C4—C5	0.1 (4)	C4—C5—C6—N2	179.2 (2)
C7—N1—C5—C4	−179.9 (2)	C6—N2—C7—N1	−1.2 (3)
C7—N1—C5—C6	0.0 (2)	C6—N2—C7—C8	176.5 (2)
C3—C4—C5—N1	−179.6 (2)	C5—N1—C7—N2	0.7 (2)
C3—C4—C5—C6	0.4 (3)	C5—N1—C7—C8	−177.0 (2)
C2—C1—C6—N2	−179.6 (2)	N2—C7—C8—C9	135.7 (2)
C2—C1—C6—C5	0.1 (3)	N1—C7—C8—C9	−47.0 (3)
C7—N2—C6—C1	−179.1 (2)	C7—C8—C9—C10	−66.3 (3)
C7—N2—C6—C5	1.2 (2)	C8—C9—C10—O1	23.1 (3)
N1—C5—C6—C1	179.5 (2)	C8—C9—C10—O2	−159.0 (2)

### Hydrogen-bond geometry (Å, °)

**Table:**

*D*—H···*A*	*D*—H	H···*A*	*D*···*A*	*D*—H···*A*
N1—H1N···O1^i^	0.86	1.86	2.700 (2)	166
N2—H2N···O2^ii^	0.86	1.80	2.654 (3)	170
O2W—H2W1···O1W^iii^	0.84 (2)	2.01 (2)	2.842 (3)	172 (3)

Symmetry codes: (i) *x*, *y*+1, *z*; (ii) *x*, −*y*+1/2, *z*+1/2; (iii) −*x*+1, *y*−1/2, −*z*+1/2.
